# In Vitro Pre-Clinical Evaluation of a Gonococcal Trivalent Candidate Vaccine Identified by Transcriptomics

**DOI:** 10.3390/vaccines11121846

**Published:** 2023-12-13

**Authors:** Shea K. Roe, Brian Felter, Bo Zheng, Sanjay Ram, Lee M. Wetzler, Eric Garges, Tianmou Zhu, Caroline A. Genco, Paola Massari

**Affiliations:** 1 Department of Immunology, Tufts University School of Medicine, Boston, MA 02111, USA; shea.roe@tufts.edu (S.K.R.); caroline.genco@tufts.edu (C.A.G.); 2 Division of Infectious Diseases and Immunology, University of Massachusetts Chan Medical School, Worcester, MA 01605, USAsanjay.ram@umassmed.edu (S.R.); 3 Section of Infectious Diseases, Boston University School of Medicine, Boston, MA 02118, USA; lwetzler@bu.edu; 4 Department of Preventive Medicine and Biostatistics, F. Edward Hebert School of Medicine, Uniformed Services University, Bethesda, MD 20814, USA; eric.garges@usuhs.edu

**Keywords:** gonorrhea, vaccine, bactericidal antibodies, adjuvants, Th1/Th2 responses

## Abstract

Gonorrhea, a sexually transmitted disease caused by *Neisseria gonorrhoeae*, poses a significant global public health threat. Infection in women can be asymptomatic and may result in severe reproductive complications. Escalating antibiotic resistance underscores the need for an effective vaccine. Approaches being explored include subunit vaccines and outer membrane vesicles (OMVs), but an ideal candidate remains elusive. Meningococcal OMV-based vaccines have been associated with reduced rates of gonorrhea in retrospective epidemiologic studies, and with accelerated gonococcal clearance in mouse vaginal colonization models. Cross-protection is attributed to shared antigens and possibly cross-reactive, bactericidal antibodies. Using a Candidate Antigen Selection Strategy (CASS) based on the gonococcal transcriptome during human mucosal infection, we identified new potential vaccine targets that, when used to immunize mice, induced the production of antibodies with bactericidal activity against *N. gonorrhoeae* strains. The current study determined antigen recognition by human sera from *N. gonorrhoeae*-infected subjects, evaluated their potential as a multi-antigen (combination) vaccine in mice and examined the impact of different adjuvants (Alum or Alum+MPLA) on functional antibody responses to *N. gonorrhoeae*. Our results indicated that a stronger Th1 immune response component induced by Alum+MPLA led to antibodies with improved bactericidal activity. In conclusion, a combination of CASS-derived antigens may be promising for developing effective gonococcal vaccines.

## 1. Introduction

*Neisseria gonorrhoeae* is the obligate human pathogen causative agent of gonorrhea, a sexually transmitted infection (STI) with over 82 million cases worldwide and more than 700,000 reported cases in the U.S. alone in 2021 [1]. Gonorrhea is a multi-faceted disease. While urethral infection is mostly symptomatic in men, leading to prompt diagnosis and treatment, infected women are often asymptomatic, and thus, treatment may be delayed, leading to significant sequelae (i.e., endometritis, PID, ectopic pregnancies and infertility) [2,3]. Furthermore, gonococcal infection in men who have sex with men (MSM), limited to the rectum or the pharynx, is also largely asymptomatic and therefore evades timely diagnosis and treatment [4]. The treatment of *N. gonorrhoeae* infections is complicated by the widespread increase in antimicrobial resistance, even to the last effective FDA-approved extended spectrum cephalosporins, exacerbating the risk of untreatable gonorrhea [5,6]. Recurring gonococcal exposure may result in some strain-specific immunity, but protective memory responses are scarce [7,8]. Disseminated gonococcal infections (DGI) and co-infections with *Chlamydia*, syphilis and HIV are also reported [9]. There is a critical need for a vaccine against gonorrhea.

Several approaches to developing a human gonococcal vaccine have been explored in clinical trials. A killed, whole-organism vaccine in a human volunteer male urethral infection model (an acute infection model, unlike chronic reproductive tract infections in women [3]) did not induce heterologous and long-term protection [10,11]. A pilin-based subunit vaccine only led to strain-specific antibody-mediated protection in a controlled male urethral challenge model due to protein’s sequence variability [12,13]. A gonococcal outer membrane (OM) vaccine enriched in gonococcal porin was thwarted by blocking antibodies against reduction-modifiable protein (Rmp) [14,15], a conserved, antigenic gonococcal surface protein present in a small quantities, and did not show a significant difference among vaccinated and placebo infected groups [16]. Other gonococcal vaccine candidates in pre-clinical development include a peptide mimic (mimotope) of a gonococcal lipooligosaccharide (LOS) epitope called 2C7 [17,18] and several surface membrane proteins, identified in vitro by conventional screening methods, reverse vaccinology [19]), “omics” and bioinformatics [20,21,22,23,24]. Several vaccine candidates have shown variable rates of success in an estradiol-treated female mouse model of gonococcal vaginal colonization using different adjuvants and delivery systems [25,26,27,28,29]. Despite their limitations, mouse models of gonococcal colonization have replicated findings related to gonococcal virulence factors in humans, for example, LOS sialylation [30] and lipid A phosphoethanolamine [31], supporting their use for vaccine preclinical analyses. Vaccination with the 2C7 peptide mimic and passive immunization with mAb 2C7 have demonstrated that protection against gonococcal challenge in mice correlates with the presence of complement-dependent bactericidal antibodies [32,33]. Although the correlates of protection against natural gonococcal mucosal infection in humans remain unclear, most vaccine studies rely on complement-dependent antibody-mediated bacterial killing (also called serum bactericidal activity, or SBA), which serves as a correlate of protective immunity to *N. meningitidis* in humans [34]. SBA is regarded as an in vitro surrogate of protection to guide the evaluation of vaccine candidates’ efficacy in vitro [35,36]. Other mechanisms of protection may include antibody-dependent opsonophagocytosis, the blocking of bacteria adhesion/invasion at the site of colonization, and T cell responses [37], although these are not all confirmed in human studies or unequivocally in experimental mouse models of gonococcal vaginal colonization. 

Interest in the use of outer membrane vesicles (OMVs) as a multi-antigen vaccine has been revitalized by the recent observation of the decreased risk of gonococcal infection in individuals vaccinated with meningococcal OMV-based vaccines. The MeNZB vaccine was reported to have 31% efficacy against gonococcal infection in a retrospective epidemiologic study of people immunized with the MenZB vaccine [38,39]. VA-MENGOC-BC- and 4CMenB (Bexsero)-vaccinated cohorts were also reported to incur in lower rates of gonorrhea [40,41,42,43,44]. Currently, there are multiple randomized clinical trials underway to evaluate the protective efficacy of existing *N. meningitidis* OMV vaccines against gonococcal infection [45]. In mice, 4CMenB accelerated the clearance of gonococcal infection and induced antibodies with bactericidal activity [46,47,48]. Cross-protection is attributed to shared meningococcal and gonococcal antigens [49,50,51]. The administration of OMVs with IL-12 as an adjuvant accelerated bacterial clearance in a mouse model of gonococcal colonization, likely due to the stimulation of protective Th1 responses and the concomitant reduction in deleterious Th17 responses [52]. Protection in mice afforded by this vaccine has also relied on antibodies, evidenced by a lack of efficacy in B cell-deficient mice [53]. Using a “collection” of antigens, such as in an OMV, may result in a more diversified functional response against multiple epitopes compared to mono-antigen vaccines [54,55,56]. 

Our previous studies using the gonococcal transcriptome expressed during natural human mucosal infection in men and women highlighted the following: (1) *N. gonorrhoeae* gene expression varies in the male and female reproductive tract environments, (2) gonococcal gene expression and regulation are different in vivo and in vitro, and (3) a large number of gonococcal genes expressed during human infection encode hypothetical proteins. Focusing on the latter, we designed a novel bioinformatics-based Candidate Antigen Selection Strategy (CASS) and identified several new potential vaccine antigens that are expressed in vivo during natural human gonococcal infection [24] (in contrast to antigens expressed in bacteria grown in vitro). Our initial studies in mice with three CASS antigens, NGO0690, NGO0948 and NGO1701, showed the robust induction of antibodies with serum bactericidal activity (SBA) against several *N. gonorrhoeae* strains in mice immunized with Alum as the adjuvant. SBA titers were increased by combining individual mouse sera, showing that the presence of bactericidal antibodies against more than one antigen enhanced the killing of *N. gonorrhoeae* [24]. Here, we expand the characterization of these antigens by verifying recognition by human serum antibodies from *N. gonorrhoeae*-infected subjects, and by investigating their potential as a multi-antigen vaccine in mouse immunization studies. Furthermore, we examined the effect of different adjuvants (Alum (Th2 adjuvant) and MPLA (Th1 adjuvant)) on the type and magnitude of functional antibody responses against *N. gonorrhoeae*. 

## 2. Materials and Methods 

### 2.1. Antigens

Expression and purification of recombinant NGO0690, NGO0948 and NGO1701 was carried out as previously described [24].

### 2.2. Immunization of Mice

Female BALB/c mice (4–6 weeks old) (Jackson Labs, Bar Harbor, ME, USA) were housed, cared for and immunized according to NIH, Tufts University, and University of Massachusetts Chan Medical School IACUC-approved protocols. Mice (*n* = 5) were immunized subcutaneously three times following a three-week schedule with recombinant NGO0690, NGO0948 and NGO1701 combined (10 µg each), adsorbed with Alum (Imject, 40 mg/mL aluminum hydroxide, 40 mg/mL magnesium hydroxide) (Thermo Fisher Scientific, Waltham, MA, USA, #77161) at a 1:1 *v*/*v* ratio, as specified by the manufacturer, in a final volume of 100 µL of antigen/adjuvant mixture. For adsorption, Alum was added dropwise to the antigens and mixed for 30 min at room temperature (R.T.) prior to use, as specified by the manufacturer. Additional mice (*n* = 10) were immunized with antigens adsorbed with Alum as above and with MPLA (10 µg/mouse/dose) (Avanti Lipids, Alabaster, AL, USA) as an adjuvant. Control groups were immunized with adjuvants alone in PBS. Before the first immunization, preimmune sera were collected, and immune sera two weeks after each immunization (weeks 2, 5 and 8). Vaginal lavages were also collected two weeks after the last immunization. All sera and lavages were stored at −80 °C. 

### 2.3. Bacterial Strains and Growth Conditions

*N. gonorrhoeae* strains F62 (Pil+/Opa+) and MS11 were plated overnight from frozen glycerol stocks on GC base agar plates containing 1% (*v*/*v*) IsoVitaleX^®^ at 37 °C with 5% CO_2_ and grown in liquid GC broth (GCB) with 1% IsoVitaleX^®^ [24]. Bacterial growth was monitored spectrophotometrically at O.D._600_. For some experiments, aliquots of bacteria suspension at O.D._600_ > 1 were formalin-killed by incubation with 1% paraformaldehyde for 1 h at 4 °C, washed and resuspended in PBS.

### 2.4. Human Sera

Banked, de-identified sera from women with disseminated gonococcal infection (DGI) [57] (*n* = 7) were provided by Dr. Peter Rice, MD, University of Massachusetts Chan Medical School. Use of the human serum was approved by the University of Massachusetts Chan Medical School IRB. The collection and use of DGI sera were approved by the Institutional Review Board (IRB) of (–at that time) the Trustee of Health and Hospitals of the City of Boston; subjects provided informed consent. We also utilized banked, de-identified sera from male (*n* = 25) and female (*n* = 25) armed service members with uncomplicated acute gonococcal infection (10–30 days following diagnosis) and convalescent sera from additional male (*n* = 25) and female (*n* = 25) subjects (180–240 days from diagnosis). These serum samples were from a collection of specimens from the Department of Defense Serum Repository: The Armed Forces Health Surveillance Branch, Defense Health Agency, Silver Spring, Maryland (serum specimen year(s) 2010–2017; specimens received 5 September 2019) provided in collaboration with COL Eric C. Garges, MD, MPH—Uniformed Services University of the Health Sciences, Department of Preventive Medicine and Biostatistics. Use of the human serum was approved by the USUHS Human Subjects Research Program Office. Human serum was collected as part of routine public health surveillance, and therefore, consent for this study was not available. However, samples provided for this work were de-identified and approved for use by the Armed Forces Health Surveillance Branch, Defense Health Agency. All banked human sera used were determined to not involve human subject research and did not require IRB approval for use in this study at Tufts University. Commercially available, pooled whole normal human serum (abbreviated as NHS) (Pel-Freez Biologicals, Rogers, AK, USA, #34019) was also used.

### 2.5. Human Antibody ELISA

ELISA plates (Immulon 4 HBX) were coated with 2 μg/mL of purified recombinant NGO0690, NGO0948, NGO1701 and native gonococcal PIB (PIB_1B_) [58] in carbonate buffer pH 9.0 (100 µL/well) or with formalin-fixed *N. gonorrhoeae* (1–1.5 × 10^8^ CFU/mL) in PBS (100 µL/well) overnight at 4 °C. Plates were washed, and blocked with 1% BSA in PBS/0.05% Tween-20 (PBS/T) for 1 h at R.T. prior to overnight incubation at 4 °C with human sera as above (1:100 dilution), followed by incubation with an AP-conjugated secondary anti-human total IgG (Southern Biotech, Birmingham, AL, USA) and 1-step PNPP (p-nitrophenyl phosphate) reagent (Thermo Fisher Scientific). O.D._405_ values were measured spectrophotometrically. Individual serum specimens were tested in triplicate and IgG levels expressed as the combined mean O.D._405_ minus the O.D._405_ of the control antigen without serum (referred to as blank throughout the Methods and Results sections) ± SD for each antigen.

### 2.6. Mouse Antibody ELISA

ELISA plates were coated with purified proteins (2 μg/mL) or formalin-fixed *N. gonorrhoeae* (1–1.5 × 10^8^ bacteria/mL) as described above. Blocking and incubations were carried out as above, using serial dilutions of pooled mouse preimmune sera, immune sera or vaginal lavages, and AP-conjugated secondary anti-mouse total IgG, IgG1, IgG2a, IgG3 or IgM antibodies (Southern Biotech). Pooled sera and vaginal lavages were tested in triplicate or quadruplicate. Total IgG, IgG1, IgG2a, IgG3 and IgM were quantified (µg/mL ± SD) using antibody reference standard curves (Southern Biotech) and a linear regression function [24]. The Th1:Th2 ratios were calculated as IgG2a (O.D._405_ − blank)/IgG1 (O.D._405_ − blank). 

### 2.7. Antibody Avidity

Avidity was measured using a chaotrope-based ELISA assay to disrupt low-avidity antigen–antibody binding [59,60]. Briefly, plates were coated with whole *N. gonorrhoeae* as above and blocked with 3% non-fat dry milk in PBS/T for 1 h at R.T., followed by incubation with serial dilutions of mouse sera overnight. The next day, plates were washed, and two duplicate sets of wells were treated with 8M Urea in PBS/T or PBS/T alone (untreated) for 5 min at R.T. Plates were washed again prior to secondary antibody incubation and detection as above. The avidity index (AI) was expressed as the average of urea (O.D._405_ − blank)/untreated (O.D._405_ − blank) values × 100 ± SD. 

### 2.8. Cytokine ELISA

Th2-type cytokines (IL-4 and IL-10), Th1-type cytokines (IL-12p70 and IFN-γ), IL-6 and TNF-α were assessed in pooled preimmune and immune mouse sera by ELISA with Opt-EIA kits (BD Biosciences, San Jose, CA, USA) per the manufacturer’s specifications. Cytokines were expressed in pg/mL ± SD and the values were used to calculate the IL-12p70/IL-10 and the IFN-γ/IL-4 ratios. 

### 2.9. Serum Bactericidal Activity (SBA)

SBA was carried out in 96-well U-bottom plates in a 75 µL total volume as previously described [24]. Commercially available IgG/IgM-depleted normal human serum (pooled serum, Pel-Freez Biologicals, #34010) was used as the source of complement. Briefly, *N. gonorrhoeae* cultures, grown as above, were diluted to an O.D._600_ of 0.2 (2–4 × 10^8^ CFU/mL), followed by serial dilution to 2–4 × 10^4^ CFU/mL. 12.5 µL of the suspension was transferred to wells containing HBSS with 0.15 mM CaCl_2_ and 1 mM MgCl_2_, in the presence or absence of 2% BSA [48]. Wells were incubated for 20 min at R.T. with serial dilutions of mouse sera that were previously heat-inactivated (56 °C for 30 min), including: whole preimmune and immune sera, and preimmune and immune sera previously depleted of IgM using an anti-mouse IgM (μ-chain specific)−Agarose conjugated antibody (Sigma-Aldrich, Louis, MO, USA, #A4540). Complement (10%) was added, and 5–10 µL aliquots of the suspension were immediately plated in triplicate on IsoVitaleX^®^-GC agar plates (Time 0). After a further incubation at 37 °C for 30 min, additional aliquots were plated in triplicate as above (Time 30). The next day, bacterial survival (a measure of killing) was determined by CFU counting. Survival was expressed as the percentage of CFUs at T30/T0 ± SD, and the bactericidal titer as the reciprocal of the lowest serum dilution with ≤50% survival after 30 min. Controls included bacteria alone and bacteria incubated with complement alone. 

### 2.10. Statistical Analysis

GraphPad Prism 10 (GraphPad Software, Inc., San Diego, CA, USA) was used to determine statistical significance using one-way or two-way analyses of variance (ANOVA) with Tukey’s multiple comparisons or with Dunnett’s tests. Statistically significant *p* values are indicated as **** *p* < 0.0001, *** *p* < 0.001, ** *p* < 0.01 or * *p* < 0.05 in the text and in Figure legends. 

### 2.11. Modeling and B Cell Epitope Predictions

Protein structure predictions of NGO0690 (hypothetical protein; NCBI Accession number WP_003691259.1; UniProtKB Q5F8S0_NEIG1), NGO0948 (outer membrane protein assembly factor BamC; NCBI Accession number WP_003693315.1; UniProtKB D6H8H8) and NGO1701 (four-helix bundle copper-binding protein; NCBI Accession number WP_003689877.1; UniProtKB Q5F665_NEIG1) were obtained with AlphaFold [61,62] based on the available protein sequences in the NCBI Reference Sequence: NC_002946. Linear (continuous) and conformational (discontinuous) B cell epitopes were predicted with ElliPro [63] using standard cut-offs (protrusion index: minimum score (S) = 0.5, maximum distance (R) (in Angstroms) = 6). Linear epitope predictions were also confirmed by BepiPred 3.0 [64] (percentage cut-off = 20, default threshold = 0.1512). Structure modeling was rendered with PyMol 2.5.4 [65].

## 3. Results

### 3.1. Antigen Recognition by Human Sera

**Figure 1:**
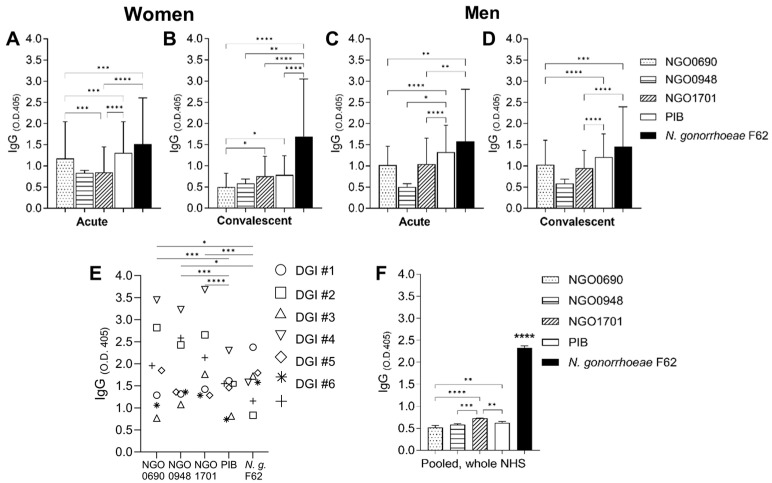
IgG against NGO0690, NGO0948 and NGO1701 in human sera from *N. gonorrhoeae*-infected subjects. Total IgG antibody ELISA of banked de-identified sera from: (**A**) women with uncomplicated acute gonococcal infection (collected 10–30 days following diagnosis) (*n* = 25); (**B**) women convalescing from uncomplicated infection (collected 180–240 days following diagnosis) (*n* = 25); (**C**) men with uncomplicated acute gonococcal infection as above (*n* = 25); and (**D**) men convalescing from uncomplicated infection as above (*n* = 25) against purified recombinant NGO0690 (dotted bars), NGO0948 (dashed bars), NGO1701 (striped bars) and *N. gonorrhoeae* F62 (black bars). Sera (1:100 dilution) were tested in triplicate or quadruplicate, and IgG levels are expressed as the mean of the IgG O.D._405_ minus the blank ± SD for each set of sera against each antigen. *, **, ***, ****—*p* value is significant according to two-way ANOVA with Tukey’s multiple comparisons test. (**E**) Banked de-identified sera from women with disseminated gonococcal infection (DGI) (*n* = 7). Individual data points are shown by different symbols. *, ***, ****—*p* value is significant according to two-way ANOVA with Tukey’s multiple comparisons test. (**F**) Commercially available pooled whole normal human serum (NHS) as above. **, ***, ****—*p* value is significant according to one-way ANOVA with Tukey’s multiple comparisons test.

The gonococcal hypothetical proteins NGO0690, NGO0948 and NGO1701 are gonococcal vaccine antigens discovered using a novel Candidate Antigen Selection Strategy (CASS) [24,66,67,68]. The immunization of mice with the individual purified proteins and Alum as an adjuvant induced the robust production of IgG that recognized several *N. gonorrhoeae* strains. Here, NGO0690, NGO0948 and NGO1701 recognition by human sera from *N. gonorrhoeae*-infected subjects was determined by ELISA. As a comparator of IgG responses, we used purified PIB porin (PorB_1B_) (from *N. gonorrhoeae* F62), an antigen known to induce an antibody response following natural infection [69], and *N. gonorrhoeae* strain F62 (whole organisms). We first examined banked de-identified sera from women (*n* = 25) and men (*n* = 25) with uncomplicated acute gonococcal infection (10–30 days from diagnosis, designated as acute sera) and banked de-identified convalescent sera from different women (*n* = 25) and men (*n* = 25) with uncomplicated infection (180–240 days from diagnosis). IgG levels in each serum were expressed as the mean O.D._405_ minus the blank. All sera recognized *N. gonorrhoeae* F62 similarly (Figure 1A–D, black bars), and the three proteins with some variability (i.e., levels of IgG against NGO0948 (Figure 1A–D, striped bars) were consistently lower than levels of IgG against NGO0690 and NGO1701) (Figure 1A–D, dotted and dashed bars, respectively). All sera also recognized *N. gonorrhoeae* PIB (Figure 1A–D, white bars). Interestingly, lower levels of IgG against the purified antigens, including PIB, were observed in convalescent sera from women compared to acute sera taken 10–30 days from diagnosis (Figure 1A,B). Because uncomplicated *N. gonorrhoeae* infection does not induce significant immune responses [70,71], we also examined banked sera from women with disseminated gonococcal infection (DGI) [57]. We reasoned that the invasive nature of DGI would elicit higher antibody responses. As shown in Figure 1E, levels of IgG against NGO0690, NGO0948 and NGO1701 in the DGI sera were higher than the corresponding IgG levels in acute or convalescent sera from both women and men with uncomplicated infection (see Figure 1A–D). Interestingly, IgG reactivity to PIB was similar across groups (Figure 1E); this could be due, in part, to the limited cross-reactivity of anti-porin antibodies in the sera (the majority of strains causing DGI express the PIB_1A_ porin allele [72]) against the *N. gonorrhoeae* PIB_1B_ porin that was used as the target in the ELISA assay. Uncomplicated infection is mostly caused by PIB_1B_ strains, which are more prevalent than PIB_1A_ among circulating strains [72]. As a control, commercially available, pooled whole normal human sera (NHS) were used, which showed lower IgG reactivity against all the purified antigens compared to the DGI sera (Figure 1F), indicating the induction of specific antibodies following invasive natural gonococcal infection in humans. Of note, normal human serum showed IgG levels comparable to post-infection sera, suggesting the presence of cross-reactive antibodies against *N. gonorrhoeae,* possibly elicited by colonization with other Neisserial species.

### 3.2. Antibody Responses in Mice to a Multi-Antigen Vaccine and Effects of Adjuvants

We previously reported that the combination of anti-NGO0690 + anti-NGO1701 mouse sera increased the killing of *N. gonorrhoeae* when compared to the respective individual antisera [24]. Here, we immunized mice with a combination of NGO0690, NGO0948 and NGO1701 to assess quantitative and qualitative antibody responses. We used Alum (Th2-biased adjuvant that induces higher IgG1 levels relative to IgG2a [73]) as a comparator to bridge our prior results, and Alum+MPLA as an adjuvant to elicit a balanced Th1 and Th2 response. MPLA (monophosphoryl lipid A) is a TLR4 adjuvant that induces preferentially Th1-skewed responses [74]. Th1 responses offer better protection against gonococcal infection than Th2-type responses in mice [52], and Th1-type antibodies (IgG2a/b) have higher complement-dependent bactericidal activity against *N. gonorrhoeae* than IgG1 [75].

#### 3.2.1. Total IgG Antibody Responses to Purified Antigens

**Figure 2:**
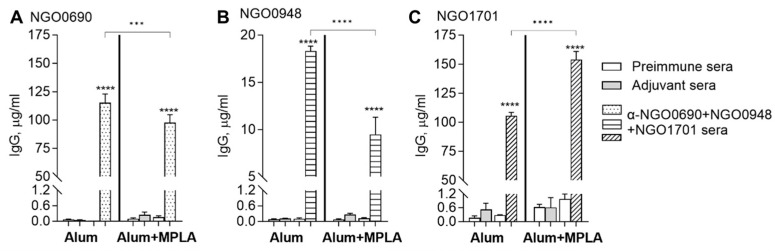
Mouse serum IgG antibodies against purified antigens. Total IgG (μg/mL ± SD) in sera from mice immunized with NGO0690+NGO0948+NGO1701 and Alum or Alum+MPLA as an adjuvant measured by ELISA against (**A**) NGO0690 (dotted bars), (**B**) NGO0948 (dashed bars) and (**C**) NGO1701 (striped bars). Preimmune sera, white bars; sera from mice immunized with adjuvant only, gray bars. Sera were tested in triplicate or quadruplicate. ***, ****—*p* value is significant according to one-way ANOVA with Tukey’s multiple comparisons test. Note the different scale in (**A**) and (**C**) vs. (**B**).

We measured total serum IgG responses elicited by immunization with NGO0690+NGO0948+NGO1701 with Alum or with Alum+MPLA as an adjuvant. The combined antigens with Alum induced higher anti-NGO0690 and anti-NGO0948 total IgG antibodies than the combined antigens with Alum+MPLA (Figure 2A,B, dotted and dashed bars, respectively), while using Alum+MPLA as an adjuvant led to higher anti-NGO1701 IgG levels (Figure 2C, striped bars). The levels of IgG against all the antigens in preimmune sera or sera from mice immunized with the adjuvant alone were very low to negligible (Figure 2A–C, white bars and gray bars, respectively). 

**Figure 3:**
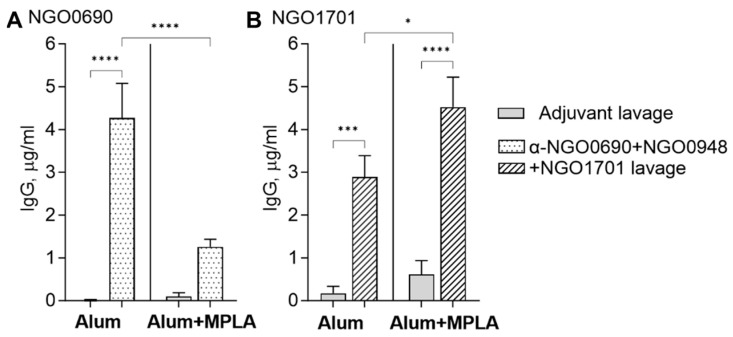
Mouse vaginal lavage IgG antibodies against purified antigens. Total IgG (μg/mL ± SD) in vaginal lavages from mice immunized with NGO0690+NGO0948+NGO1701 and Alum or Alum+MPLA as an adjuvant measured by ELISA against (**A**) NGO0690 (dotted bars) and (**B**) NGO1701 (striped bars). Lavages from mice immunized with adjuvant only, gray bars. Lavages were tested in quadruplicate. *, ***, ****—*p* value is significant according to one-way ANOVA with Tukey’s multiple comparisons test.

The IgG levels in the mouse vaginal lavages paralleled the serum levels, with antigens mixed with Alum inducing higher anti-NGO0690 IgG (Figure 3A, dotted bars), and antigens with Alum+MPLA inducing higher anti-NGO1701 IgG levels (Figure 3B, striped bars). Consistent with the observed low anti-NGO0948 IgG levels in sera, vaginal lavage IgG antibodies against NGO0948 were also low (0.054 µg/mL with Alum and 0.0098 µg/mL with Alum+MPLA as an adjuvant). The differences in the immunogenicity of each antigen may be related to their nature or structure (NGO0690 and NGO0948 are predicted lipoproteins [24]) or, possibly, interactions with the adjuvant [76].

#### 3.2.2. Serum and Vaginal Lavage Total IgG Induced by Combined Antigens against Whole *N. gonorrhoeae*

**Figure 4:**
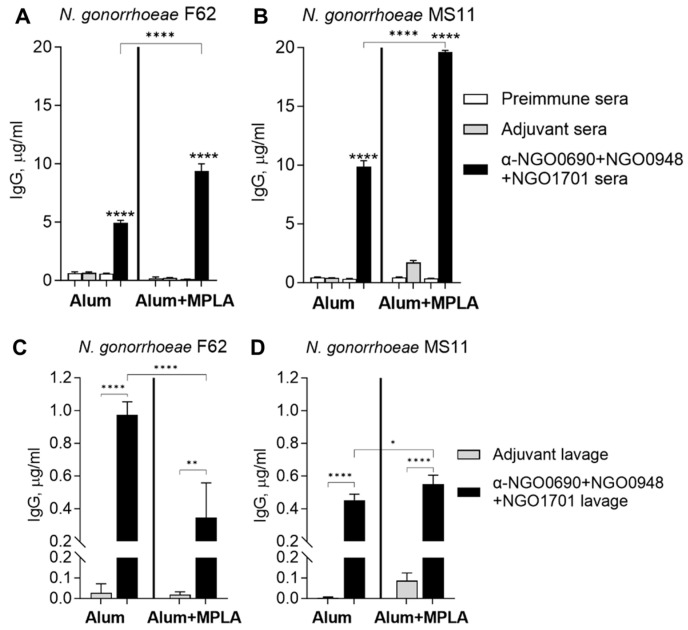
Mouse serum IgG antibodies against whole *N. gonorrhoeae*. Total IgG (μg/mL ± SD) in sera from mice immunized with NGO0690+NGO0948+NGO1701 and Alum or Alum+MPLA, measured by whole-cell ELISA against (**A**) *N. gonorrhoeae* F62 (black bars) and (**B**) *N. gonorrhoeae* MS11 (black bars). Preimmune sera, white bars; sera from mice immunized with adjuvant only, gray bars. (**C**,**D**) Total IgG in vaginal lavages from mice immunized with NGO0690+NGO0948+NGO1701 and Alum or Alum+MPLA, measured as above. Lavages from mice immunized with adjuvant only, gray bars. Sera and lavages were tested in triplicate or quadruplicate. *, **, ****—*p* value is significant according to one-way ANOVA with Tukey’s multiple comparisons test.

The immune IgG recognition of the *N. gonorrhoeae* strains F62 and MS11 was assessed by whole-cell ELISA. Immunization with NGO0690+NGO0948+NGO1701 adjuvanted with Alum+MPLA induced higher total IgG against both strains than the Alum-adjuvanted antigens (Figure 4A,B, black bars). Low, non-specific reactivity to *N. gonorrhoeae* strains was observed in preimmune sera and sera from mice immunized with either adjuvant alone (Figure 4A,B, white and gray bars, respectively). Mouse vaginal lavage IgG against *N. gonorrhoeae* F62 induced by the combined antigens and Alum were higher than when using Alum+MPLA (Figure 4C, black bars), while similar vaginal lavage IgG were detected against *N. gonorrhoeae* MS11, regardless of the adjuvant used (Figure 4D, black bars). However, vaginal lavage levels of IgG against whole *N. gonorrhoeae* were lower overall than to the purified antigens (See Figure 3). Vaginal lavage IgG induced by immunization with the adjuvant alone were low to negligible (Figure 4C,D, gray bars).

#### 3.2.3. Serum IgG Antibody Subclasses against Whole *N. gonorrhoeae*

**Figure 5:**
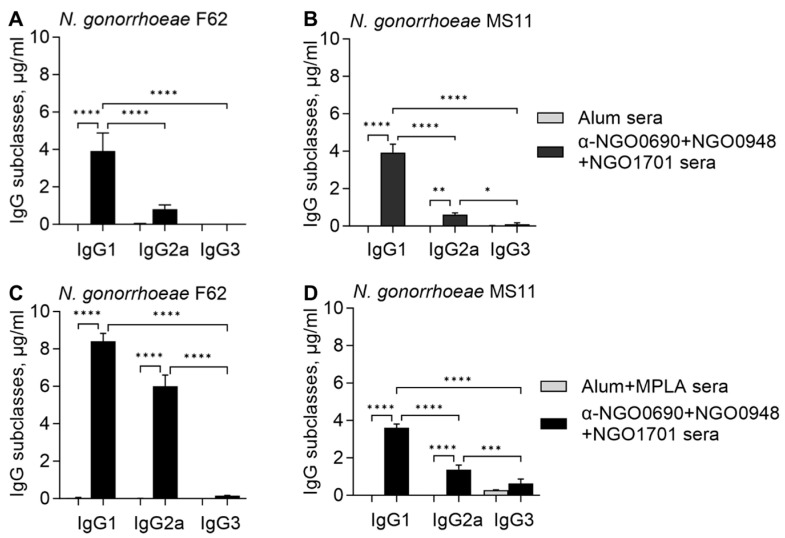
Mouse serum IgG antibody subclasses against whole *N. gonorrhoeae*. IgG1, IgG2a and IgG3 (μg/mL ± SD) measured by whole-cell ELISA in sera from mice immunized with NGO0690+NGO0948+NGO1701 with Alum (**A**,**B**) or with Alum+MPLA (**C**,**D**) as adjuvants against (**A**,**C**) *N. gonorrhoeae* F62 (black bars) and (**B**,**D**) *N. gonorrhoeae* MS11 (black bars). Preimmune sera, white bars; adjuvant-only sera, gray bars. Sera were tested in triplicate or quadruplicate. *, **, ***, ****—*p* value is significant according to one-way ANOVA with Tukey’s multiple comparisons test.

The IgG subclasses’ responses to *N. gonorrhoeae* were examined by whole-cell ELISA, and as expected, immunization with NGO0690+NGO0948+NGO1701 and Alum elicited primarily IgG1, low levels of IgG2a and lower amounts of IgG3 antibodies that recognized *N. gonorrhoeae* F62 and MS11 (Figure 5A,B, black bars). In contrast, immunization with combined antigens using Alum+MPLA induced higher IgG1 and IgG2a levels that reacted with *N. gonorrhoeae* F62 (Figure 5C, black bars), and higher IgG2a and IgG3 against *N. gonorrhoeae* MS11 (Figure 5D, black bars). The Th1:Th2 antibody subclass ratio (IgG2a/IgG1) indicated a stronger Th1 component (IgG2a/IgG1 ratio approaching 1) when MPLA was added to Alum (Table 1) compared to Alum alone (IgG2a/IgG1 ratio less than 0.5). These results confirmed that the addition of MPLA to Alum promoted a stronger Th1-biased antibody response.

### 3.3. Serum Cytokine Production Induced by Combined Antigens

**Figure 6:**
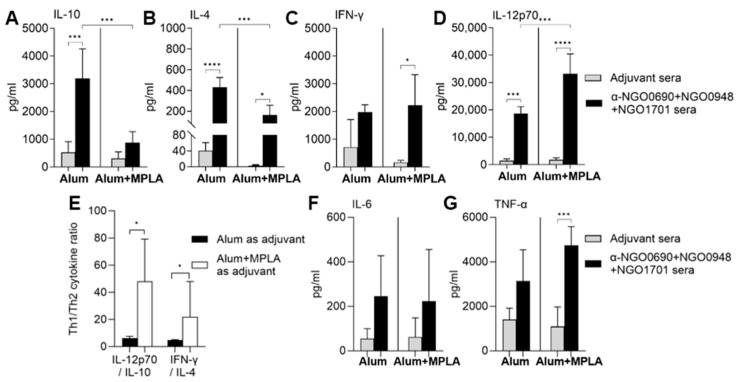
Mouse serum cytokine profile. Th2 cytokines (**A**) IL-10 and (**B**) IL-4, and Th1 cytokines (**C**) IFN-γ and (**D**) IL-12p70 measured by ELISA. Adjuvant-only sera (gray bars), NGO0690+NGO0948+NGO1701 and Alum sera and NGO0690+NGO0948+NGO1701 and Alum+MPLA (black bars) were tested in quadruplicate and cytokine levels are expressed in pg/mL ± SD. *, ***, ****—*p* value is significant according to one-way ANOVA with Tukey’s multiple comparisons test. (**E**) IL-12p70/IL-10 ratio and IFN-γ/IL-4 ratio. * *p* < 0.05 according to Mann–Whitney test. (**F**,**G**) IL-6 and TNF-α measured as above. ***—*p* value is significant according to one-way ANOVA with Tukey’s multiple comparisons test.

Serum cytokines were examined by ELISA to establish a more complete view of the (cellular in addition to humoral) response elicited by immunization with NGO0690+NGO0948+NGO1701 with Alum or Alum+MPLA. Immunization with Alum induced significantly higher IL-4 and IL-10 (Th2-type cytokines) levels than Alum+MPLA (Figure 6A,B, black bars), and Alum+MPLA induced significantly higher IL-12p70 than Alum (Figure 6D, black bars), while INF-γ levels were similar using either adjuvant (Figure 6C). As a result, IFN-γ/IL-4 and IL-12p70/IL-10 ratios were significantly higher when Alum+MPLA was used as an adjuvant (Figure 6E). Together with the IgG subclass results, the serum cytokine profiles supported a stronger Th1-biased response when Alum+MPLA was used as an adjuvant. The induction of IL-6 and TNF-α was comparable when using either adjuvant (Figure 6F,G). 

### 3.4. Serum IgM Antibodies against Whole N. gonorrhoeae Induced by Combined Antigens

**Figure 7:**
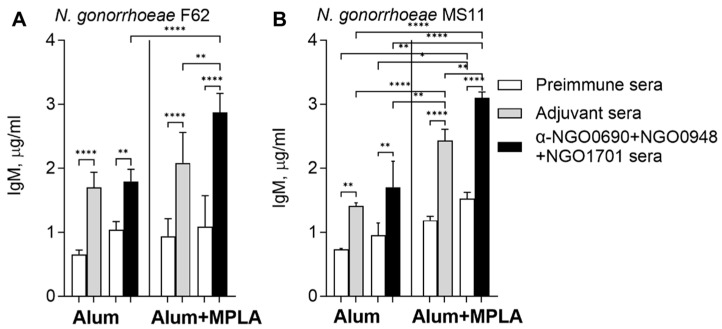
Mouse serum IgM antibodies against whole *N. gonorrhoeae*. IgM (μg/mL ± SD) in sera from mice immunized with NGO0690+NGO0948+NGO1701 and Alum or Alum+MPLA, measured by whole-cell ELISA against (**A**) *N. gonorrhoeae* F62 (black bars) and (**B**) *N. gonorrhoeae* MS11 (black bars). Preimmune sera, white bars; adjuvant-only sera, gray bars. Sera were tested in triplicate or quadruplicate. *, **, ****—*p* value is significant according to one-way ANOVA with Tukey’s multiple comparisons test.

IgM antibodies that reacted with *N. gonorrhoeae* were measured by whole-cell ELISA. Natural IgM against *N. gonorrhoeae* F62 and MS11 were detected in preimmune mouse sera (Figure 7A,B, white bars), and were increased in sera from mice immunized with the adjuvant alone (Figure 7A,B, gray bars). Pre-existing cross-reactive IgM antibodies may increase with advancing age in mice or via a non-specific effect of the adjuvants. There was a small but statistically significant increase in IgM levels that recognized both gonococcal strains in sera from mice immunized with NGO0690+NGO0948+NGO1701 and Alum+MPLA compared to Alum (Figure 7A,B, black bars). 

### 3.5. Antibody Avidity

**Figure 8:**
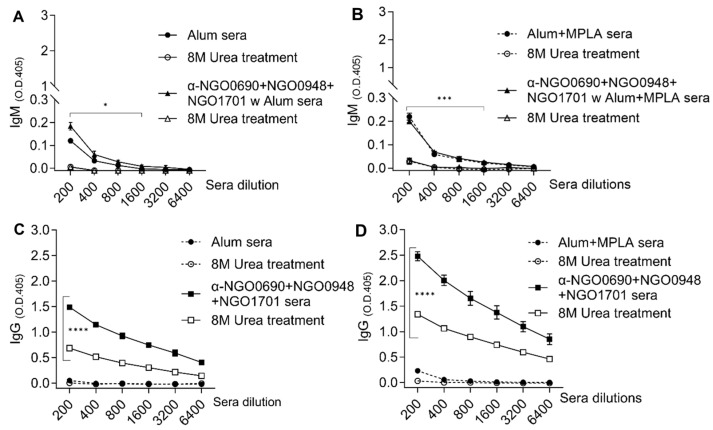
Serum IgM and IgG antibody avidity against *N. gonorrhoeae* F62. (**A**) IgM (O.D._405_ minus the blank ± SD) determined with a modified ELISA in the presence (open symbols) or absence (closed symbols) of 8M urea treatment. NGO0690+NGO0948+NGO1701 and Alum sera (triangles) or Alum-alone sera (circles) and (**B**) NGO0690+NGO0948+NGO1701 and Alum+MPLA sera (triangles) or Alum+MPLA-alone sera (circles). (**C**) IgG antibody avidity as above. Alum-alone sera (circles) and NGO0690+NGO0949+NGO1701 with Alum sera (squares) and (**D**) Alum+MPLA-alone sera (circles) and NGO0690+NGO0949+NGO1701 with Alum+MPLA sera (squares). Sera were tested in triplicate. *, ***, ****—*p* value is significant according to two-way ANOVA with Tukey’s multiple comparisons test.

Serum IgG and IgM antibody specificity for *N. gonorrhoeae* was evaluated by measuring antibody avidity in a modified ELISA using the chaotropic agent urea to disrupt low-affinity antigen–antibody binding. IgM binding to *N. gonorrhoeae* F62 in sera from mice immunized with NGO0690+NGO0948+NGO1701 and either Alum or Alum+MPLA was low, similar to IgM binding in sera from adjuvant-only-immunized mice (Figure 8A,B, closed triangles and circles, respectively). Treatment with urea (8M) disrupted IgM antibody binding (Figure 8A,B, open triangles and circles), consistent with the low avidity of murine natural and elicited IgM against *N. gonorrhoeae*. At higher serum concentrations, there was a small statistically significant difference in IgM binding. The avidity index (AI) (percentage of antibodies bound to the antigen after treatment with urea) of IgM was low (≤10) for all sera [77]. A similar low AI was also observed for IgG antibodies in sera from adjuvant-only immunized groups in the presence or absence of urea treatment (Figure 8C,D, closed and open circles). In contrast, immunization with NGO0690+NGO0948+NGO1701 and either adjuvant induced IgG with high avidity, shown by the partial disruption of IgG-*N. gonorrhoeae* binding by treatment with urea (Figure 8C,D, closed and open squares). The AI for IgG antibodies was 41.2 and 50.3, respectively. 

The analysis of the IgG subclass avidity showed that immunization with NGO0690+NGO0948+NGO1701 and either Alum or Alum+MPLA induced IgG1 antibodies with comparably high avidity for *N. gonorrhoeae* F62 (AI of 53 and 67, respectively), and that Alum+MPLA induced IgG2a antibodies with higher avidity than Alum (AI of 67 vs. 26, respectively). 

### 3.6. Serum Bactericidal Activity

**Figure 9:**
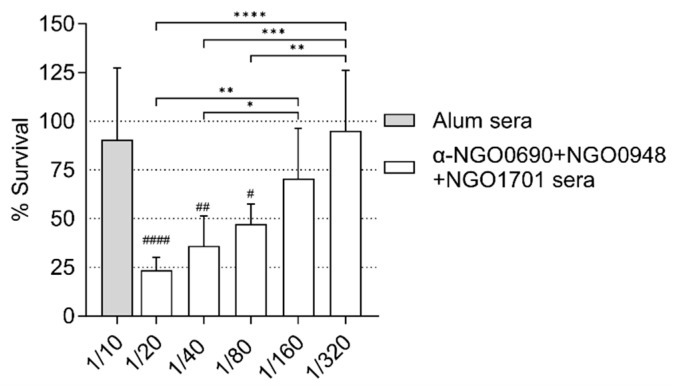
Serum bactericidal activity (SBA). *N. gonorrhoeae* F62 survival (% CFU at T30/T0 ± SD). IgM-depleted sera from mice immunized with Alum alone (gray bar) and NGO0690+NGO0948+NGO1701 with Alum (white bars). Serum dilutions are indicated on the *X*-axis. ^#, ##, ####^—*p* < 0.05, 0.003 and 0.0001 according to one-way ANOVA with Dunnett’s multiple comparison test vs. the Alum-only sera. *, **, ***, ****—*p* = 0.04 to <0.0001 according to one-way ANOVA with Tukey’s multiple comparisons test among sera dilutions.

The complement-mediated antibody-dependent bactericidal activity (SBA) of sera from mice immunized with NGO0690+NGO0948+NGO1701 and Alum was examined against *N. gonorrhoeae* F62 using IgM-depleted mouse sera. The bactericidal titers (i.e., the highest serum dilution that resulted in <50% survival of bacteria after 30 min. incubation with mouse sera and IgG/IgM-depleted pooled human sera as a source of complement) were about 1/80 (Figure 9, white bars). These titers were comparable to our previous results with anti-NGO0690 sera and anti-NGO1701 sera combined [24]. Bacterial killing was not observed by incubation with IgG/IgM-depleted human complement alone or with non-heat-inactivated mouse sera, as previously shown [24] or with IgM-depleted sera from mice immunized with Alum alone (Figure 9, gray bar). 

IgM depletion avoids the interference of SBA by natural (or induced) bactericidal IgM antibodies in mouse sera. As an alternative to IgM depletion, the addition of 2% BSA in the SBA has been shown to efficiently block killing by nonspecific or low-binding antibodies [48]. Indeed, whole mouse sera from the Alum-only-immunized group killed *N. gonorrhoeae*, but live bacteria were rescued in the presence of 2% BSA (Supplemental Figure S1A,B, gray bars). In contrast, the SBA of sera from mice immunized with NGO0690+NGO0948+NGO1701 and Alum was only slightly decreased in the presence of 2% BSA (bacterial survival increased from 4.5% to 14% in a 1/10 serum dilution) (Supplemental Figure S1A,B, white bars). These results indicate that the bactericidal activity of nonspecific (or natural) IgM antibodies against *N. gonorrhoeae* is blocked by 2% BSA.

**Figure 10:**
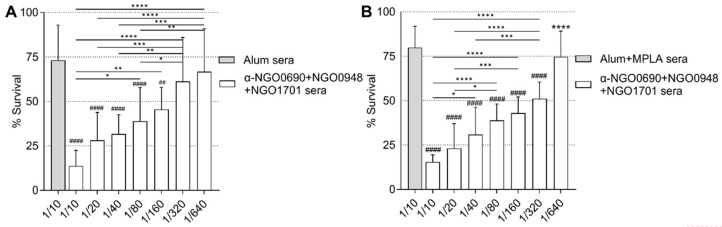
Serum bactericidal activity (SBA) in the presence of 2% BSA. *N. gonorrhoeae* F62 survival (% CFU T30/T0 ± SD). Sera from mice immunized with (**A**) Alum alone (gray bar) or with NGO0690+NGO0948+NGO1701 and Alum (white bars), and (**B**) Alum+MPLA-alone (gray bar) or NGO0690+NGO0948+NGO1701 and Alum+MPLA (white bars). Serum dilutions are indicated on the *X*-axis. *, **, ***, ****—*p* = 0.04 to <0.0001 according to one-way ANOVA with Tukey’s multiple comparisons test among sera dilutions. ^##^, ^####^—*p* = 0.002 and <0.0001 according to one-way ANOVA with Tukey’s multiple comparisons test vs. adjuvant alone.

Using these assay conditions, the SBA titers of sera from mice immunized with NGO0690+NGO0948+NGO1701 and Alum remained about 1/80–1/160 overall (Figure 10A), similar to the IgM-depleted SBA results in the absence of 2% BSA (Figure 9). The SBA titers of sera from mice immunized with NGO0690+NGO0948+NGO1701 and Alum+MPLA were ~2-fold higher (about 1/160–1/320) (Figure 10B). It is possible that the higher amount of IgG2a antibody against *N. gonorrhoeae* induced by using Alum+MPLA as an adjuvant (~6-fold higher than when using Alum alone; see Figure 5) and their higher avidity to *N. gonorrhoeae* contribute to the observed increase in SBA.

## 4. Discussion

There is an urgent need for a vaccine against *N. gonorrhoeae* infection. Currently, several potential candidate vaccines are being investigated, including individual antigens and outer membrane vesicles options [78,79]. The latter offers the opportunity to induce a diverse pool of antibodies that recognize multiple antigens. Previous work from our group has identified new gonococcal hypothetical proteins as potential vaccine candidates via a novel Candidate Antigen Selection Strategy (CASS) that combines an analysis of the gonococcal transcriptome during natural human mucosal infection and immunobioinformatics [24]. Three candidates, selected by CASS, NGO0690, NGO0948 and NGO1701, induced robust bactericidal antibody responses in mice. Immunogenicity in mice is important for studying potential vaccine candidates, but antigen recognition by human immune responses is crucial. Because our antigens were identified through an analysis of human natural mucosal infection specimens, it is likely that the corresponding proteins are expressed by the gonococcus during infection. However, although the three target antigens showed comparable levels of mRNA expression in samples from both infected men and women, the actual levels of expressed proteins during infection are unknown. If expression is low, if a protein is not easily accessible, or if it is not very immunogenic in humans, antibodies may be quantitatively or qualitatively insufficient and not recognize the antigen in vivo. Using sera from naturally infected humans, we showed the presence of IgG antibodies against NGO0690, NGO0948 and NGO1701 in women and men with uncomplicated gonorrhea. A comparative analysis of our sera from acute infections and convalescent sera (non-longitudinal samples) showed variability in levels of IgG antibodies against some antigens in convalescent sera from infected women (NGO0690, NGO0948 and PIB). It remains unclear whether this might be due to declining antibody responses, and its significance could only be expanded in studies using longitudinal samples. Sera from women with DGI showed higher IgG levels, consistent with the induction of antibodies resulting from the systemic nature of this disease [70]. The levels of antibodies induced against the three antigens were similar overall, suggesting that they were sufficiently expressed by *N. gonorrhoeae* during natural infection and were immunogenic in humans. Differences in the levels of antibodies against PIB and *N. gonorrhoeae* F62 in the DGI sera may be attributable to differences in the porin type and the original infecting strains in this small number of samples. Of note, IgG in commercially available human sera from uninfected subjects reacted strongly with *N. gonorrhoeae* F62 but showed low levels of IgG against the antigens. 

Although immune correlates of protection against *N. gonorrhoeae* in humans have not been defined, complement-dependent antibody-mediated bacterial killing (serum bactericidal activity, SBA) may be considered a surrogate of protection against vaginal colonization in preclinical vaccine evaluation [36,48]. For at least two vaccine antigens, the 2C7 LOS mimotope and chimeric antigen comprising NGO0265 plus FtsN [33,80], evidence suggests that SBA may constitute a mechanistic correlate of protection against gonococcal vaginal colonization of mice. Bactericidal antibodies are also induced by the immunization of mice with 4CMenB [46,48], although no studies have correlated this with protection in vivo in mice yet, nor have they determined whether a strong SBA translates to higher protection. However, although SBA cannot be considered the only surrogate of protection, it is a valuable in vitro tool to move forward with preclinical analyses of potential vaccine candidates prior to testing them all in mouse models of vaginal colonization. We previously reported that a combination of anti-NGO0690 and anti-NGO1701 mouse sera had greater bactericidal activity than the individual antisera [24]. We sought to expand these results by using NGO0690, NGO0948 and NGO1701 as a multi-component vaccine candidate. Multivalent vaccines against bacterial and viral pathogens, and even cancer, may elicit broader and possibly better protection than monovalent vaccines; this is especially true for bacterial vaccines where antigen expression varies among strains [54,55,56]. An example is the meningococcal OMV vaccine, where the addition of NadA (Neisserial adhesin A) and fHbp (factor H binding protein) results in a synergistic increase in (*N. meningitidis*) killing antibodies [54,81,82]. Targeting multiple epitopes also raises the bar for the development of vaccine resistance, because pathogens would have to alter several epitopes, each potentially important for virulence, to escape the vaccine. Thus, ‘vaccine-resistant’ mutants may be attenuated in vivo because of compromised fitness and/or virulence [54]. We also evaluated adjuvants that skew immune responses differently. Alum, which we used previously, is a Th2-skewed adjuvant that induces predominantly IgG1 antibodies [73]. Protection from gonococcal infection mostly correlates with Th1 responses in mice and in humans (whereas Th17 responses are non-protective) [52,53,83,84]; Th1-biased antibody subclasses, IgG2a and IgG2b, activate complement better than IgG1, and potentiate serum bactericidal activity against *N. gonorrhoeae* [75]. For this reason, we added MPLA to Alum in an attempt to enhance Th1 responses. Alum and MPLA are components of the AS04 adjuvant licensed for use in human vaccines (Fendrix and Cervarix [85]), which could also potentially be used for a gonococcal vaccine. 

We reported an overall increase in IgG responses to individual antigens and to whole *N. gonorrhoeae* in sera from mice immunized with NGO0690+NGO0948+NGO1701 and Alum+MPLA compared to using only Alum. Specific IgG, but not IgA, were also detected in vaginal lavages, likely due to the systemic immunization route used. Although serum antibody responses may not necessarily reflect mucosal responses, IgG are dominant in vaginal secretions in humans, because they are transported across epithelial cells by FcRn [86,87]. IgG in human vaginal fluid may permit complement-dependent bacterial killing in the reproductive tract because a hemolytically active complement system is present in cervical secretions [88]. Serum antibody binding to purified antigens was greater than to whole bacteria, possibly related to the actual amount of protein captured in the microtiter wells, but also perhaps to diminished epitope exposure on and/or antibody access to whole organisms. Lastly, the differences in antibody production to a given protein with different adjuvants may be attributable to antigen–adjuvant interactions [76]. The analysis of the antibody subclasses indicated that adding MPLA to Alum induced a shift towards a more robust Th1 antibody component (IgG2a), although IgG1 remained the predominant subclass due to the effect of Alum. Cytokine identification, which also represents a surrogate marker for Th1 and Th2 skewing, confirmed that the addition of MPLA to Alum resulted in higher IL-12 and INF-γ and lower IL-10 and IL-4 levels than Alum alone. IL-12 and IL-10 have antagonistic and interdependent functions [89], and their balance suggests that MPLA mitigated the strong Th2 bias of Alum.

IgM antibodies that recognized whole *N. gonorrhoeae* organisms were also detected in mouse preimmune sera, likely non-specific natural IgM (produced by B-1a cells in mice [90]). IgM levels were increased by immunization with the adjuvant alone, which could be due to aging of the mice or, in the case of MPLA, to an effect of MPLA itself via TLR4 signaling [85,91]. Antigen-specific low-avidity IgM were also induced by NGO0690+NGO0948+NGO1701 with both adjuvants. As expected, IgG elicited by the adjuvants alone had low avidity for *N. gonorrhoeae*, which contrasted with the high-avidity-specific IgG elicited by NGO0690+NGO0948+NGO1701 adjuvanted with Alum or Alum+MPLA. While Alum induced only IgG1 with high avidity, the addition of MPLA also led to the induction of IgG2a with high avidity, supporting specificity and more prominent skewing towards a Th1 antibody response. 

The bactericidal activity of mouse antisera raised with NGO0690+NGO0948+NGO1701 and Alum was examined using IgM-depleted sera or sera treated with 2% BSA, two methods that allow for the exclusion of the potential killing of *N. gonorrhoeae* by natural pathogen-binding IgM in whole mouse sera [92]. In both cases, the SBA titers in immune sera were between 1/80 and 1/160, consistent with titers previously reported using a combination of sera from mice immunized with individual antigens (NGO0690 and NGO1701) [24]. When using Alum+MPLA as an adjuvant, the SBA titers were ~1/160–1/320, suggesting that by increasing Th1-type responses, the addition of MPLA may also have an effect on SBA. Antibody avidity and bactericidal activity correlate [59,60,93], as do antibody avidity and protection by the meningococcal and HiB vaccines, where low-avidity antibodies are cited as one of the causes for limited success in infants [94,95]. Whether or not there is a similar relationship between IgG avidity and gonorrhea vaccine efficacy remains unclear, but we speculate that a vaccine that induces high concentrations of high-avidity antibodies with robust SBA titers, accompanied by a Th1-skewed profile, that recognize multiple gonococcal strains is desirable. Other antibody-mediated functions frequently examined in vitro include opsonophagocytic killing and the inhibition of host cell adhesion/invasion, all of which may be impacted by vaccination with a multi-antigen vaccine composed of outer membrane/periplasmic proteins. Targeting multiple epitopes in several antigens may also increase strain coverage. 

**Figure 11:**
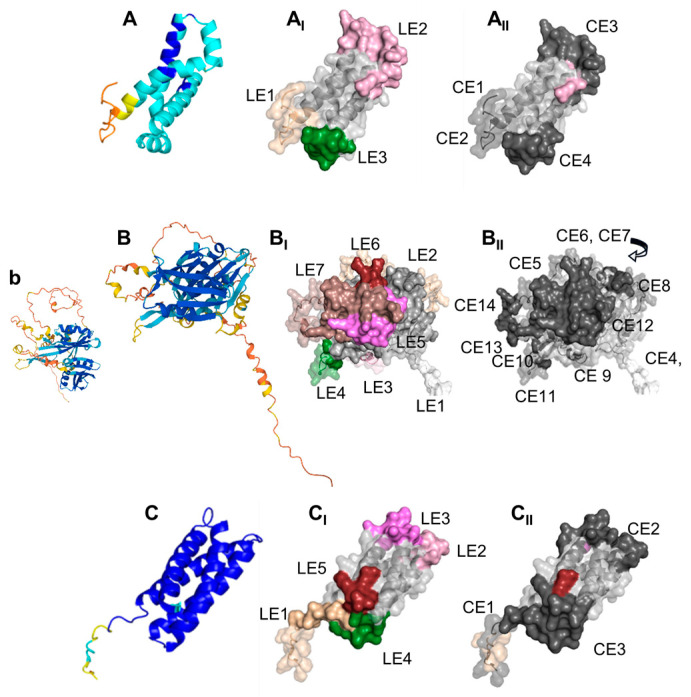
Images of the predicted structure (side view) of (**A**) NGO0690, (**B**) NGO0948 and (**C**) NGO1701, modeled with Alpha Fold [61], based on the protein sequence and colored by confidence (dark blue, highest; orange/yellow, lowest). (**b**) NGO0948, front view. (**A_I_**–**C_I_**) Surface model images of NGO0690, NGO0948 and NGO1701 (light gray) rendered with PyMol [65], showing the predicted linear B cell epitopes (LE) (ElliPro and Bepipred [63,64]) in different colors. LE in regions with low structure model confidence as in (**A**–**C**) is shown by transparent colors, and LE in regions with high confidence in solid colors. (**A_II_**–**C_II_**) Predicted conformational epitopes (CE) obtained and rendered as above. CE in regions of low confidence are shown in transparent dark gray, and CE in regions with high confidence in solid dark gray. In (**B_II_**), CE6 and CE7 are at the back of the image, indicated by an arrow, and are not visible.

Thus, the intelligent design of a combination subunit vaccine may be an effective mechanism to induce varied protective responses. This could be achieved either by using individual antigens combined, or possibly by generating chimeric antigens, as recently shown for two gonococcal candidates discovered via a machine learning platform [80]. Should a gonococcal OMV-based vaccine be pursued, an additive approach could be taken, as for the meningococcal 4CMenB vaccine, using CASS antigens or other candidates. On the other hand, some caveats that should be considered for expanding the number of antigens in a subunit vaccine, or possibly even in an OMV vaccine, include potential antagonism between antibodies, which could diminish the effect of otherwise protective antigens in the challenge model (as previously seen with Rmp, a target for blocking Ab) [96]. Existing challenges in the production of recombinant (CASS or other) antigens by conventional methods, particularly outer membrane proteins, could be eased by expression in non-bacterial systems and cell-free systems. As an alternative to individual or multi-antigen subunit vaccines, peptide-based synthetic vaccines (epitope vaccines) could also be designed to include B cell and T cell peptides individually or as chimeric antigens corresponding the individual targets. The images in Figure 11 show (1) the predicted structure of (A) NGO0690, (B) NGO0948 and (C) NGO1701 colored by confidence (darker blue, highest confidence; orange/yellow, lowest confidence); (2) the predicted linear epitopes (LE) (Figure 11A_I_–C_I_) and (3) the predicted conformational epitopes (CE) (Figure 11A_II_–C_II_) for each protein. The amino acid sequence of the predicted peptides is shown in Supplementary Tables S1–S3, along with the full protein sequences. Our ongoing exploration of additional CASS antigens will eventually further enrich the pool of potential new gonococcal vaccine candidates for future testing in vivo. 

## Figures and Tables

**Table 1:** Th1:Th2 ratio (IgG2a (O.D._405_ − blank)/IgG1 (O.D._405_ − blank) ± SEM).

Adjuvant Group	*N. gonorrhoeae* F62	*N. gonorrhoeae* MS11
Alum	0.35 ± 0.02 ^a^	0.31 ± 0.006 ^a^
Alum+MPLA	0.93 ± 0.02 ^b^	0.51 ± 0.02

^a^ *p* < 0.001 vs. Alum+MPLA and ^b^ *p* < 0.0001 vs. *N. gonorrhoeae* MS11 according to one-way ANOVA with Tukey’s multiple comparison test.

## Data Availability

The data presented in this paper are available on request from the corresponding author.

## Supplementary Materials

The following supporting information can be downloaded at: https://www.mdpi.com/article/10.3390/vaccines11121846/s1, Supplementary Figure S1: Serum bactericidal activity (SBA). Supplementary Table S1: NGO0690-predicted linear (LE) and conformational (CE) epitopes. Supplementary Table S2: NGO0948-predicted linear (LE) and conformational (CE) epitopes. Supplementary Table S3: NGO1701-predicted linear (LE) and conformational (CE) epitopes.
