# Supplementary material for: In Vitro Pre-Clinical Evaluation of a Gonococcal Trivalent Candidate Vaccine Identified by Transcriptomics
Source: Vaccines (Basel). 2023 Dec 13;11(12):1846. doi: 10.3390/vaccines11121846 (PMC10747275; doi:10.3390/vaccines11121846)
Supplement: Supplementary file 1 [file vaccines-11-01846-s001.zip › Roe et al - Revised Suppl Tables.pdf]

Supplemental Table S1. NGO0690 predicted linear (LE) and conformational (CE) epitopes

NGO0690

>WP_003691259.1 hypothetical protein [Neisseria gonorrhoeae]
MNKTLSILPAAILLGGCAAGGNTFGSLDGGTGMGGSIVKMTVESQCRAELDRRSEWRLTALAMSAEKQAEWENKICGCATEEAPNQLTGND
VMQMLNQSTRNQALAALTVKTVSACFKRLYR

n.	Start	End	Sequence	Residues	ElliPro Score	Color *
**Linear Epitopes (LE)**						
1	20	33	GGNTFGSLDGGTGM	14	0.8	Trsp Wheat
2	52	70	RRSEWRLTALAMSAEKQAE	19	0.7	Lt Pink
3	93	100	MQMLNQST	8	0.64	Green
**Conformational epitopes (CE)**						
1			G20-T23	4	0.94	Trsp Dk Gray
2			F24-M33	10	0.75	Trsp Dk Gray
3			R53-E55, R57-E70	17	0.74	Dk Gray
4			L87-N90, M93-Q94, L96-T100, Q103	12	0.67	Dk Gray

Gray-highlighted residues in the sequence above: predicted signal peptide, not shown in the cartoon models in **Figure 11A, 11A$_{I}$** and **11A$_{II}$**

Dashed underlined: Low confidence regions predicted by AlphaFold (yellow / orange in **Figure 11A**)

* Epitope color in **Figure 11A$_{I}$ and A$_{II}$**

Supplemental Table S2. NGO0948 predicted linear (LE) and conformational (CE) epitopes

NGO0948

```
>WP_003693315.1 outer membrane protein assembly factor BamC [Neisseria gonorrhoeae]
MPSEPFGRHNMTNTLISITQDDTMTHIKPVIAALALIGLAACSGSKTEQPKLDYQSRSHRLIKLEVPPDLNNPDQGNLYRLPAGSGAVRASDLE
KRRTPAVQQPADAEVLKSVKGVRLERDGSQRWLVVDGKSPAEIWPLLKAFWQENGFDIESEEPAIGQMETEWAENRAKIPQDSLRRLFDTVGLG
GIYSTGERDKFIVRIEQGKNGVSDIFFAHKAMKEVYGDKNKDTTMWQPSASDPNLEAAFLTRFMQYLGVDGRQAENALAKKPTLPAANEMARIE
GKSLIVFGDYGRNWRRTGLALDRIGLTVVGQNTERHAFLVQKAPNESNAVTEQKPGLFKRLLGKGKAEKPAEQPELIVYAEPVADGSRIVLLNK
DGSAYAGKDASALLGKLHSELR
```

Linear epitopes (LE)

n.	Start	End	Sequence	Residues	ElliPro Score	Color *
1	1	44	MPSEPFGRHNMTNTLISITQDDTMTHIKPVIAALALIGLAACSG	44	0.87	Trsp White
2	61	115	LIKLEVPPDLNNPDQGNLYRLPAGSGAVRASDLEKRRTPAVQQPADAEVLKSVKG	55	0.74	Trsp Wheat
3	173	192	IPQDSLRRLFDTVGLGGIYS	20	0.68	$ Trsp Lt Pink
4	224	233	YGDKNKDTTM	10	0.71	Trsp Green
5	280	292	RIEGKSLIVFGDY	13	0.58	Dk Pink
6	314	319	NTERHA	6	0.63	Ruby
7	331	387	AVTEQKPGLFKRLLGKGKAEKPAEQPELIVYAEPVADGSRIVLLNKDGSAYAGKDAS	57	0.66	Taupe

Conformational epitopes (CE)

n.	Sequence	Residues	ElliPro Score	Color *
1	M1-T19	19	0.97	-
2	Q20-H26	7	0.94	-
3	P29-A40, C42-G44	15	0.72	Trsp White
4	L61-I62, L64	3	0.58	Trsp Dk Gray
5	E65-Y79	15	0.82	Trsp Dk Gray
6	L81-K95, T98-L110	28	0.74	Trsp $$ Dk Gray
7	K111-K114, *G256*	5	0.56	Dk Gray$$
8	G115, *D130-S133, E136*, *G206-V210*	11	0.53	Dk Gray
9	I173-P174, D176-L178, L181-F182	7	0.64	Dk Gray $
10	D183-Y191	9	0.74	Dk Gray
11	Y224-M233, *S329*, *N330*-Q335	17	0.62	Dk Gray
12	R280-S285, I287, F289-Y292, N314-A319, K351-I359, Y361-R370, V372-S387	52	0.63	Dk Gray
13	K336-K341	6	0.79	Dk Gray $
14	L343-E350	8	0.80	Dk Gray $

Gray-highlighted residues in the sequence: predicted putative signal peptide, only shown in the cartoon model in **Figure 11b and 11B.**

Dashed underlined: Low confidence regions predicted by AlphaFold (yellow / orange in **Figure 11B**)

* Epitope color in **Figure $11B_{I}$ and $11B_{II}$**

$ Partial view in the cartoon model in **Figure $11B_{II}$**

$$ Not visible in the cartoon model in **Figure $11B_{II}$**

Italic: Residues not predicted in LE

Supplemental Table S3. NGO1701 predicted linear (LE) and conformational (CE) epitopes

NGO1701
>WP_003689877.1 four-helix bundle copper-binding protein [Neisseria gonorrhoeae]
MNRRQFLGSAAAVSLASAASFARAHGHADYHHHHDMQPAAASAYTAVRQTAAHCLDAGQVCLTHCLSLLTQGDTSMSDCAVAVRQMLALCGAV
HDLAAQNSPLTRDAAKVCLEACKQCAKACKEHSAHHAECKACYESCLDCIKECEKLAA

n.	Start	End	Sequence	Residues	ElliPro Score	Color *
			**Linear epitopes (LE)**			
1	25	44	HGHADYHHHHDMQPAAASAY	20	0.82	Wheat
2	63	78	THCLSLLTQGDTSMSD	16	0.74	Lt Pink
3	99	106	QNSPLTRD	8	0.63	Green
4	123	133	KEHSAHHAECK	11	0.62	Dk Pink
5	147	151	EKLAA	5	0.75	Ruby
			**Conformational epitopes (CE)**			
1			H25-H31	7	0.96	Trsp Dk Gray
2			Q37-Y44, Q99-L103, R105-D106, *K109*, K149-A151	20	0.68	Dk Gray
3			T63-D78, K123-E131, K133, *E137*	27	0.70	Dk Gray

Gray-highlighted residues in the sequence: predicted signal peptide, not shown in the cartoon models in **Figure 11C, 12C$_{I}$ and 11C$_{II}$**
Dashed underlined: Low confidence regions predicted by AlphaFold (yellow / orange in **Figure 11C**)
* Epitope color in **Figure 11C$_{I}$ and 11C$_{II}$**
Italic: Residues not predicted in LE
