# Supplementary figures and images for: In Vitro Pre-Clinical Evaluation of a Gonococcal Trivalent Candidate Vaccine Identified by Transcriptomics
Source: Vaccines (Basel). 2023 Dec 13;11(12):1846. doi: 10.3390/vaccines11121846 (PMC10747275; doi:10.3390/vaccines11121846)

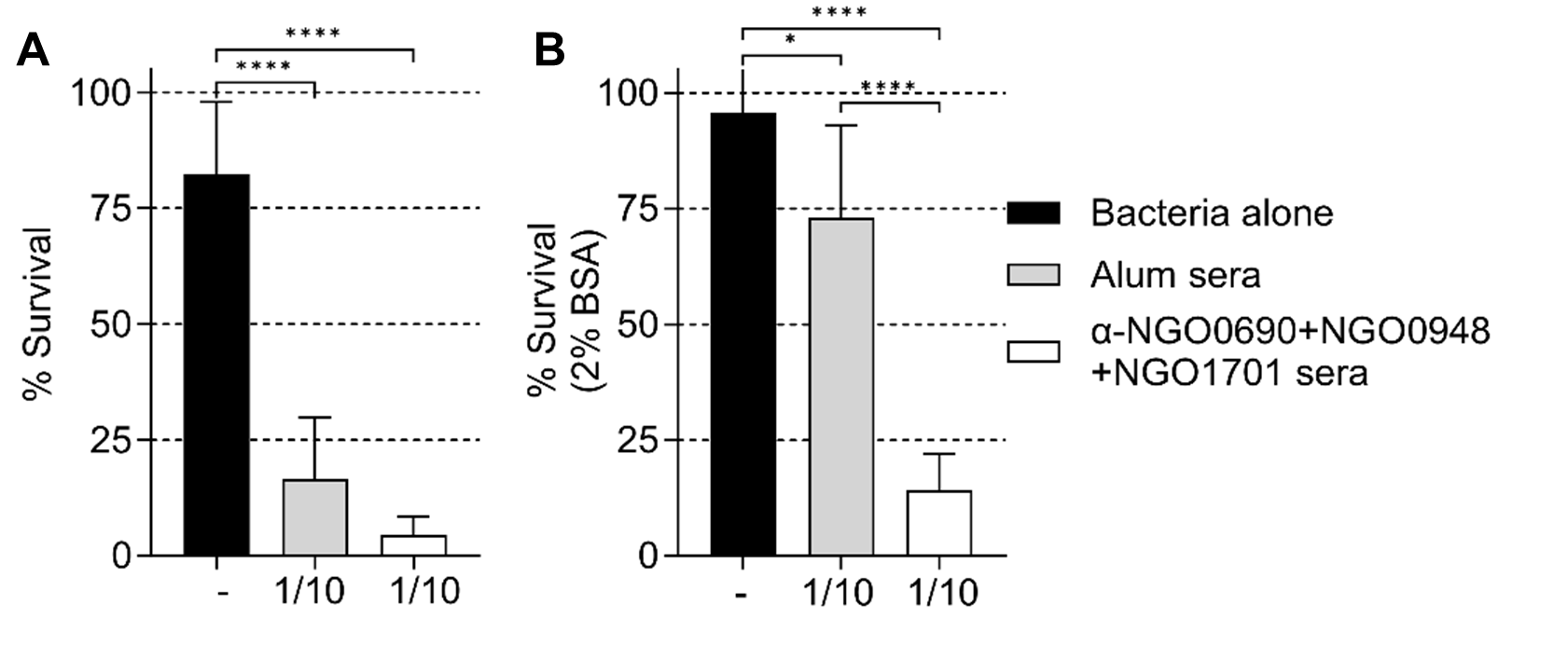

Supplement: Supplementary file 1 [file vaccines-11-01846-s001.zip › Supplemental Figure S1.tif]
